# Association of estimated glomerular filtration rate and all-cause mortality in acute pancreatitis: a retrospective analysis

**DOI:** 10.1186/s12871-023-02074-4

**Published:** 2023-04-13

**Authors:** Fang Gong, Quan Zhou, Chunmei Gui, Shaohua Huang

**Affiliations:** 1 Department of Intensive Care Unit, the First People’s Hospital of Changde, 818 Renmin Road, Changde City, 415000 Hunan China; 2 Department of Science and Education Section, the First People’s Hospital of Changde, Changde, China

**Keywords:** Acute pancreatitis, All-cause mortality, Estimated glomerular filtration rate, MIMIC-III Database, Intensive care units

## Abstract

**Background:**

Our primary objective was to explore the association between estimated glomerular filtration rate (eGFR) and all-cause mortality in acute pancreatitis (AP) admission to intensive care units.

**Methods:**

This study is a retrospective cohort analysis based on the Medical Information Mart for Intensive Care III database. The eGFR was calculated based on Chronic Kidney Disease Epidemiology Collaboration equation. Cox models with restricted cubic spline functions were used to evaluated the association of eGFR with all-cause mortality.

**Results:**

The mean eGFR was 65.93 ± 38.56 ml/min/1.73 m^2^ in 493 eligible patients. 28-day mortality was 11.97% (59/ 493), which decreased by 15% with every 10 ml/min/1.73 m^2^ increase in eGFR. The adjusted hazard ratio (95% confidence interval) was 0.85 (0.76–0.96). A non-linear association was proved between eGFR and all-cause mortality. When eGFR < 57 ml/min/1.73 m^2^, there was a negative correlation between eGFR and 28-day mortality, hazard ratio (95% CI) was 0.97 (0.95, 0.99). The eGFR was also negatively correlated with in-hospital and in-ICU mortality. Subgroup analysis confirmed that the association between eGFR and 28-day mortality in different characteristics was stable.

**Conclusions:**

The eGFR was negatively correlated with all-cause mortality in AP when eGFR is less than the threshold inflection point.

## Introduction

Acute pancreatitis (AP) is a common gastrointestinal inflammatory disorder, which is costly and difficult to manage, and there is no pathogenetic treatment [1, 2]. Severe AP may develop in 20% of patients, with a reported mortality rate of 30% due to pancreatic necrosis and organ failure [3–5].

The estimated glomerular filtration rate (eGFR) based on patient demographics and serum creatinine are recommended as the best comprehensive index to estimate renal function more accurately [6–8]. The Kidney Disease: Improving Global Outcomes (KDIGO) clinical practice guidelines [9] recommend the Chronic Kidney Disease Epidemiology Collaboration (CKD-EPI) equation [10] to be used to calculate eGFR. This equation was validated using participants with and without kidney disease and across a wide range of measurements with GFR [10]. Previous studies have confirmed that patients with lower eGFR (< 30 ml/ min/ 1.73 m^2^) have 6 to 9 times higher mortality than patients with normal eGFR in total hip arthroplasy [11]. Among a large cohort of insulin-treated type 2 diabetes patients, eGFR was associated with the greatest risk of death [12]. However, only few studies have supported the effect of eGFR on mortality in patients with AP.

In the present study, our main purpose was to explore the relationship between eGFR and 28-day all-cause mortality in AP patients in the intensive care units (ICUs), and the secondary purpose was to study the relationship between eGFR and in-hospital and ICU mortality.

## Materials and methods

### Data source

The data were extracted from the Medical Information Mart for Intensive Care version III (MIMIC-III V1.4) according to the ethical standards of the Institutional Review Board of the Massachusetts Institute of Technology. We applied the Protecting Human Research Participants test (No. 39742301). The databases cover 61,532 ICU admissions of 46,476 patients between 2001 and 2012 at the Beth Israel Deaconess Medical Center in Boston, MA [13]. MIMIC-III includes hourly physiological readings from bedside monitors, records of demographics, and other clinical data.

Diagnostic criteria for acute pancreatitis (AP) are based on the fulfilment of two of three criteria: (1) abdominal pain consistent with acute pancreatitis (2) serum lipase activity (or amylase activity) at least three times the upper limit of normal; (3) characteristic findings of acute pancreatitis on contrast-enhanced computed tomography (CECT) and less commonly magnetic resonance imaging (MRI) or abdominal ultrasonography [14]. Diagnoses of patients were recorded via the International Classification of Diseases, Ninth Revision (ICD-9) codes. Data were obtained through the Structured Query Language (SQL) performed in the MIMIC-III database.

This study was approved by the Institutional Review Boards of Beth Israel Deaconess Medical Center (Boston, MA) and the Massachusetts Institute of Technology (Cambridge, MA). This study was reported according to STROBE guidelines [15].

### Study population

A total of 961 patients with AP were retrieved according to the ICD-9 code for the diagnosis of AP (5770). Only patients at the first admission to the ICU and those aged ≥ 18 years were included. ICU length of stay < 24 h or length of hospital stay > 90 days; no ICU information; incomplete (> 10% of values missing), incorrect, or uninterpretable data; patients lacking serum creatinine; and those with metastatic tumors, solid tumors, lymphoma, or acquired immunodeficiency syndrome were excluded.

Baseline characteristics collected in the first 24 h of admission into the ICU included sex, age, comorbidities, Sequential Organ Failure Assessment (SOFA) score, and Systemic Inflammatory Response System (SIRS) score for patients with AP. Laboratory results, including white blood cell count (WBC), blood urea nitrogen (BUN), and serum creatinine were all recorded within 24 h. When the above indicators had multiple results within 24 h, we considered only the worst value. The primary outcome was 28-day mortality, and the secondary outcomes were in-hospital and ICU mortality.

### Measurement of eGFR

The independent variable in this study was eGFR, which was calculated according to the CKD-EPI equation [10] expressed as follows:

$$\mathrm{GFR}=141\times\min{(\mathrm{Scr}/\kappa,1)}^{\alpha}\times\max{(\mathrm{Scr}/\kappa,1)}^{-1.209}\times{0.993}^{\mathrm{Age}}\times1.018\lbrack\text{if female}\rbrack\times1.159\lbrack\text{if of African descent}\rbrack,$$

where Scr is serum creatinine in mg/dL, κ is 0.7 for females and 0.9 for males, α is -0.329 for females and -0.411 for males, min indicates the minimum of Scr/κ or 1, and max indicates the maximum of Scr/κ or 1.

### Statistical analysis

First, baseline data of patients included in this study were presented according to eGFR levels. Continuous variables were expressed as mean (standard deviation [SD]) when normally distributed or median (range) when non-normally distributed. Categorical variables were expressed as percentages. One-way analysis of variance (ANOVA) (normal distribution) and the χ^2^ or Fisher’s exact test (categorical variables) were used to calculate the differences among different eGFR groups, which were divided according to the clinical cut-off point.

Second, Cox proportional hazards regression models were used to evaluate the association between eGFR and 28-day all-cause mortality. In the crude model, no covariates were adjusted, and in Model I, only age and sex were adjusted. In model II, we adjusted for covariates associated with eGFR and mortality. Confounders were selected based on existing literature and clinical judgment. But also, if the influence of a covariate changed by > 10%, it would be incorporated into the adjusted model [16]. To verify the results, we conducted eGFR groups as categorical variables and further explored the possibility of non-linearity. The non-linearity between eGFR and 28-day all-cause mortality was explored using a Cox proportional hazard regression model with a restricted cubic spline and smooth curve fitting.

To test the robustness of our results, we performed a sensitivity analysis. Interaction and stratified analyses were conducted according to sex, age (< 60 and ≥ 60 years), alcohol consumption (non-drinkers and drinkers), hypertension, diabetes, congestive heart failure, chronic pulmonary disease, alcohol consumption, and cardiac arrhythmia. All analyses were performed using R 3.3.2 (http://www.R-project.org, The R Foundation) and Empower Stats (http://www.empowerstats.com, X&Y Solutions, Inc, Boston, MA, USA). Statistical significance was set at *p* < 0.05.

## Results

### Participants and baseline characteristics

**Fig. 1:**
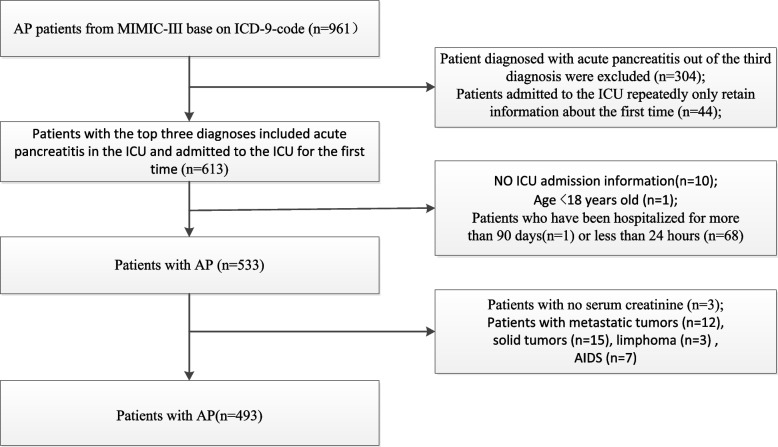
Flowchart of subject screening. Abbreviation: AP, acute pancreatitis; MIMIC-III, Medical Information Mart for Intensive Care version III; ICU, intensive care unit

A total of 961 patients with AP were reviewed according to the ICD-9 code.We excluded 468 participants who did not meet the inclusion criteria. A total of 493 participants of AP were included in the study. Figure 1 describes the inclusion and exclusion processes of the study population. The demographic and clinical characteristics of participants by levels of eGFR are summarized in Table 1. Of the 493 included participants, 231 were female (46.86%) and 262 (53.14%) were male. The mean age of participants was 60.15 ± 17.49 years. Age, BUN, creatinine, SOFA score, in-hospital mortality, in-ICU mortality, and ICU 28-day mortality were significantly higher in patients with eGFR < 30 ml/min/1.73 m^2^ than in those with eGFR ≥ 90 ml/min/1.73 m^2^.

**Table 1:** Baseline characteristics of participants by levels of estimated glomerular filtration rate (eGFR)

Characteristics		Levels of eGFR (ml/min/1.73 m^2^)				
	Overall	< 30	30–60	60–90	≥ 90	*P*-value
	*N* = 493	*N* = 118	*N* = 111	*N* = 106	*N* = 158	
Demographics						
Age	60.15 (17.49)	67.80 ± 18.31	68.10 ± 13.58	62.06 ± 15.71	47.58 ± 12.85	< 0.001
Sex						0.112
Female	231 (46.86%)	64 (54.24%)	45 (40.54%)	54 (50.94%)	68 (43.04%)	
Male	262 (53.14%)	54 (45.76%)	66 (59.46%)	52 (49.06%)	90 (56.96%)	
Clinical features, N (%)						
Congestive heart failure						< 0.001
No	391 (79.31%)	86 (72.88%)	79 (71.17%)	85 (80.19%)	141 (89.24%)	
Yes	102 (20.69%)	32 (27.12%)	32 (28.83%)	21 (19.81%)	17 (10.76%)	
Cardiac arrhythmias						< 0.001
No	342 (69.37%)	72 (61.02%)	69 (62.16%)	70 (66.04%)	131 (82.91%)	
Yes	151 (30.63%)	46 (38.98%)	42 (37.84%)	36 (33.96%)	27 (17.09%)	
Hypertension						< 0.001
No	218 (44.22%)	37 (31.36%)	45 (40.54%)	44 (41.51%)	92 (58.23%)	
Yes	275 (55.78%)	81 (68.64%)	66 (59.46%)	62 (58.49%)	66 (41.77%)	
Chronic pulmonary disease						0.26
No	420 (85.19%)	102 (86.44%)	88 (79.28%)	92 (86.79%)	138 (87.34%)	
Yes	73 (14.81%)	16 (13.56%)	23 (20.72%)	14 (13.21%)	20 (12.66%)	
Diabetes						0.051
No	352 (71.40%)	76 (64.41%)	78 (70.27%)	73 (68.87%)	125 (79.11%)	
Yes	141 (28.60%)	42 (35.59%)	33 (29.73%)	33 (31.13%)	33 (20.89%)	
Alcohol consumption						0.739
No	361 (73.23%)	90 (76.27%)	83 (74.77%)	75 (70.75%)	113 (71.52%)	
Yes	132 (26.77%)	28 (23.73%)	28 (25.23%)	31 (29.25%)	45 (28.48%)	
Blood biochemistry, mean (SD)						
eGFR ml/min/1.73 m^2^	65.93 (38.56)	16.07 ± 7.17	46.48 ± 8.64	73.97 ± 9.50	111.44 ± 15.68	< 0.001
Creatinine, mg/dl	1.10 (0.20–16.20)	3.42 ± 2.12	1.45 ± 0.33	0.98 ± 0.20	0.68 ± 0.17	< 0.001
BUN, mmol /L	28.37 (22.21)	53.19 ± 27.56	31.54 ± 14.12	20.19 ± 8.25	13.09 ± 6.66	< 0.001
WBC, 10^9/L	15.67 (7.72)	15.83 ± 8.14	16.58 ± 8.45	16.04 ± 7.05	14.66 ± 7.24	0.211
SOFA score, mean (SD)	4.83 (3.56)	7.86 ± 3.99	4.92 ± 2.78	4.00 ± 2.81	3.05 ± 2.59	< 0.001
SIRS score, mean (SD)	3.09 (0.86)	3.04 (0.97)	3.10 (0.86)	3.16 (0.81)	3.08 (0.82)	0.783
RRT, N (%)						< 0.001
No	474 (96.15%)	103 (87.29%)	110 (99.10%)	103 (97.17%)	158 (100.00%)	
Yes	19 (3.85%)	15 (12.71%)	1 (0.90%)	3 (2.83%)	0 (0.00%)	
ICU 28-day mortality, N (%)						< 0.001
No	434 (88.03%)	87 (73.73%)	98 (88.29%)	98 (92.45%)	151 (95.57%)	
Yes	59 (11.97%)	31 (26.27%)	13 (11.71%)	8 (7.55%)	7 (4.43%)	
In-hospital mortality, N (%)						< 0.001
No	428 (86.82%)	83 (70.34%)	98 (88.29%)	97 (91.51%)	150 (94.94%)	
Yes	65 (13.18%)	35 (29.66%)	13 (11.71%)	9 (8.49%)	8 (5.06%)	
In-ICU mortality, N (%)						< 0.001
No	445 (90.26%)	90 (76.27%)	103 (92.79%)	100 (94.34%)	152 (96.20%)	
Yes	48 (9.74%)	28 (23.73%)	8 (7.21%)	6 (5.66%)	6 (3.80%)	

*eGFR* Estimated glomerular filtration rate, *SD* Standard deviation, *RRT* Renal replacement therapy, *BUN* Blood urea nitrogen, *WBC* White blood cell, *SOFA score* Sequential Organ Failure Assessment score, *SIRS, score* Systemic inflammatory response syndrome, *ICU* Intensive care unit

### Association between eGFR and all-cause mortality

Table 2 shows that 28-day mortality was negatively correlated with eGFR in the crude model (non-adjusted model) and full adjusted models. The hazard ratio (HR) and 95% confidence interval (CI) was 0.80 (0.73, 0.86) in the crude model when eGFR increased by 10 ml/min/1.73 m^2^. In model I, which was adjusted for age and sex, the HR (95% CI) was 0.82 (0.75, 0.90). In model II, after adjusting for age, sex, hypertension, diabetes mellitus, congestive heart failure, chronic pulmonary disease, alcohol consumption, cardiac arrhythmias, BUN, WBC, and the SIRS score, the HR (95% CI) was 0.85 (0.76, 0.96).

**Table 2:** Relationship between eGFR and 28-day mortality in different Cox proportional hazards regression models

Exposure	Crude Model		Model I		Model II	
	HR (95%CI)	*P*-value	HR (95%CI)	*P*-value	HR (95%CI)	*P*-value
eGFR per increase 10 ml/min/1.73m^2^	0.80 (0.73, 0.86)	< 0.0001	0.82 (0.75, 0.90)	< 0.0001	0.85 (0.76, 0.96)	0.008
eGFR groups						
≥ 90	Ref		Ref		Ref	
60–90	2.58 (0.79, 8.37)	0.115	1.78 (0.54, 5.87)	0.345	2.16 (0.64, 7.23)	0.214
30–60	4.70 (1.60, 13.81)	0.005	2.66 (0.86, 8.21)	0.088	2.60 (0.82, 8.21)	0.104
< 30	9.13 (3.19, 26.10)	< 0.0001	5.82 (1.96, 17.26)	0.002	4.96 (1.41, 17.36)	0.012
*P* for trend		< 0.0001		< 0.0001		0.010

Crude Model: Adjusted none

Model I: Adjusted for gender; age

Model II: Adjusted for age, gender, hypertension, diabetes mellitus, congestive heart failure, chronic pulmonary disease, alcohol consumption, cardiac arrhythmias, BUN, WBC, and the SIRS score

*eGFR* Estimated glomerular filtration rate, *HR* Hazard ratio, *Ref* Reference, *CI* Confidence interval, *BUN* Blood urea nitrogen, *WBC* White blood cell, *SIRS score* Systemic inflammatory response syndrome

For the purpose of sensitivity analysis, we converted eGFR into a categorical variable using clinical cut-off points using multivariate proportional hazards regression models and calculated *P* for trend. The higher eGFR (≥ 90 ml/min/1.73 m^2^) group was used as the reference group, and the risk of 28-day all-cause mortality significantly increased in the lower eGFR (< 30 ml/min/1.73 m^2^) group after adjusting for potential covariates. The adjusted HR (95% CI) was 4.96 (1.41, 17.36) (*P* = 0.012). The *P* value for the trend was 0.010.

### Non-linear association between eGFR and all-cause mortality

**Fig. 2:**
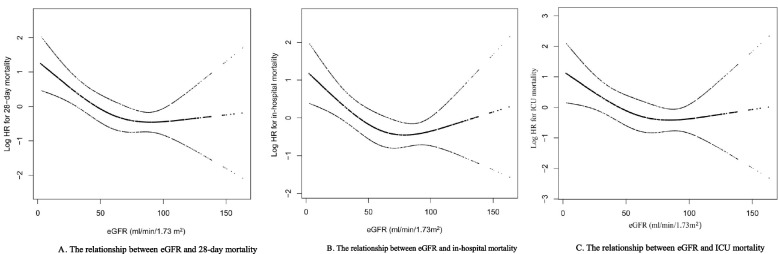
The nonlinear relationship between eGFR and all-cause mortality, including 28-day mortality (**A**), in-hospital mortality (**B**) and in-ICU mortality (**C**)

Figure 2 shows the non-linear association between eGFR and 28-day mortality (A), eGFR and in-hospital mortality (B), and eGFR and in-ICU mortality (C) by a Cox proportional hazard regression model with restricted cubic spline and smooth curve fitting. We adjusted for age, sex, hypertension, diabetes mellitus, congestive heart failure, chronic pulmonary disease, alcohol consumption, cardiac arrhythmias, BUN, WBC, and SIRS score. We further calculated the threshold inflection point as 57 ml/min/1.73 m^2^ using a two-piecewise regression model in Table 3. There was a negative correlation between eGFR and 28-day all-cause mortality on the left of the threshold inflection point, the HR (95% CI) was 0.97 (0.95, 0.99), *p* < 0.0034. However, there was no significant association on the right of the inflection point (HR 1.00; 95% CI 0.98–1.02; *p* = 0.8003).

**Table 3:** Threshold effect analysis of the relationship between eGFR and 28-day mortality using a two-piecewise regression model

eGFR inflection point	OR (95% CIs)	*p*-value
< 57 ml/min/1.73m^2^	0.97 (0.95, 0.99)	0.0034
≥ 57 ml/min/1.73m^2^	1.00 (0.98, 1.02)	0.8003
P for log likelihood ratio test		0.049

Adjusted for age, gender, hypertension, diabetes mellitus, congestive heart failure, chronic pulmonary disease, alcohol consumption, cardiac arrhythmias, BUN, WBC, and the SIRS score

*eGFR* Estimated glomerular filtration rate, *OR* Odds ratio, *Ref* Reference, *CI* Confidence interval, *BUN* Blood urea nitrogen, *WBC* White blood cell, *SIRS score* Systemic inflammatory response syndrome

### eGFR and 28-day all cause mortality

**Fig. 3:**
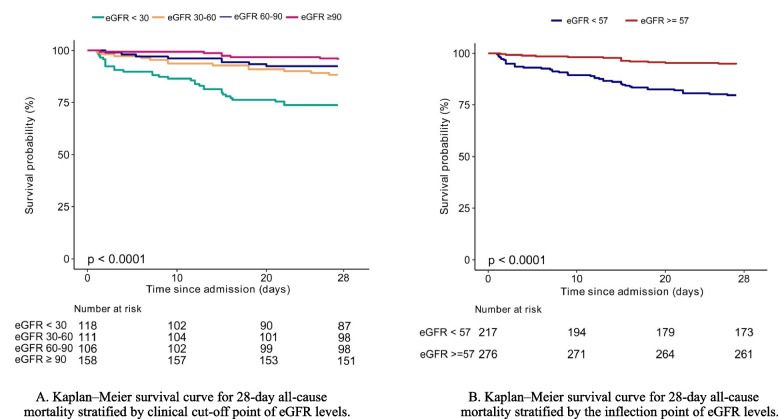
Kaplan–Meier survival curve for 28-day all-cause mortality. Kaplan–Meier survival curve for 28-day all-cause mortality stratified by clinical cut-off point of eGFR levels (**A**) and by inflection point (**B**) respectively

Figure 3 shows the Kaplan–Meier survival curve for 28-day all-cause mortality stratified by the clinical cut-off point of eGFR levels (A) and by the inflection point of eGFR (B). The curves of lower eGFR groups (< 30 ml/min/1.73 m^2^ and < 57 ml/min/1.73 m^2^) separated early and continued to diverge throughout the 28-day follow-up. The risk of 28-day mortality in the groups with eGFR < 30 ml/min/1.73 m^2^ and < 57 ml/min/1.73 m^2^ was significantly higher than in the other groups (log-rank test *P* value < 0.0001).

We further determined that in-hospital and in-ICU mortality were both negatively correlated with eGFR using Logistic regression models (Table 4). When eGFR decrease by 10 ml/min/1.73 m^2^, the OR (95% CI) of in-hospital mortality was 1.19 (1.05, 1.36) (*P* = 0.0076) and the OR (95% CI) of in-ICU mortality was 1.25 (1.06, 1.47) (*P* = 0.0066) after adjusting for age, sex, hypertension, diabetes mellitus, congestive heart failure, chronic pulmonary disease, alcohol consumption, cardiac arrhythmias, BUN, WBC, and SIRS scores.

**Table 4:** Logistic regression models according to eGFR (per decrease 10 ml/min/1.73m^2^)

Variable	Crude Model		Adjusted Model	
	OR (95% CI)	*p*-value	OR (95% CI)	*p*-value
In-ICU mortality	1.29 (1.17, 1.43)	< 0.0001	1.25 (1.06, 1.47)	0.0066
In-hospital mortality	1.27 (1.17, 1.39)	< 0.0001	1.19 (1.05, 1.36)	0.0076

Crude Model: Adjusted none

Adjusted model: Adjusted for age, gender, hypertension, diabetes mellitus, congestive heart failure, chronic pulmonary disease, alcohol consumption, cardiac arrhythmias, BUN, WBC, and the SIRS score

*eGFR* Estimated glomerular filtration rate, *HR* Hazard ratio; Confidence interval; *BUN* Blood urea nitrogen, *WBC* White blood cell

**Fig. 4:**
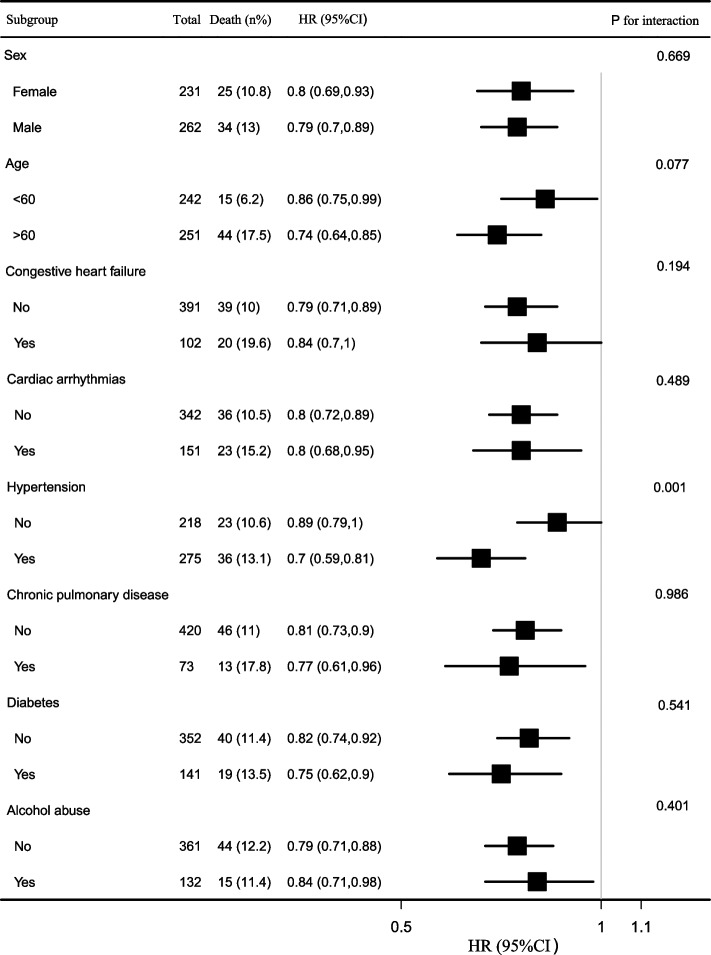
The subgroup analysis between eGFR and 28-day all-cause mortality

The subgroup analysis (Fig. 4) further verified the robustness of the results. As shown in Fig. 4, the 28-day all cause mortality was negatively correlated with eGFR in different characteristics.The change in the population with hypertension was more obvious (*P* for interaction = 0.001, HR: 0.7 with hypertension vs. 0.89 with no-hypertension).

## Discussion

In this study, we found a non-linear association between eGFR and all-cause mortality. The 28-day mortality, ICU mortality, and in-hospital mortality were all negatively correlated with eGFR. Participants with lower eGFR (< 30 ml/min/1.73 m2) were more likely to have higher mortality from all causes over time. In addition, subgroup analysis showed a stable association between eGFR and 28-day all-cause mortality in patient with AP in different characteristics.

The data is consistent with previous studies. Pal Tod and their research team report that the average eGFR was significantly lower in died patients, compared to survived patients [17]. Michal Lipinski et al. [18] found that the first-day serum creatinine and eGFR were good predictors of death in acute pancreatitis—the area under receiver operating characteristic (ROC) curve (AUC) respectively 0.879 and 0.787. They further confirmed that mortality and the presence of pancreatic necrosis were significantly higher (*p* < 0.001) in patients with lower eGFR. In a prospective, multi-national, observational cohort study [19], which included 6983 emergency departments patients, it was proved that a decrease in eGFR was significantly associated with 30-day mortality and in-hospital mortality, for 10 ml/min/1.73m^2^ decrease in eGFR the OR for the 30-day mortality was 1.15(95% CI 1.09 to 1.22, *p* < 0.001). However, these studies are only limited to comparison between groups, lacking regression analysis and adjusting for confounding factors.Our survey not only carried out regression analysis by adjusting confounding factors, but also further explored the linear and nonlinear relationship between eGFR and mortality. Our data demonstrated a higher mortality risk for the group with eGFR < 30 ml/min/1.73 m^2^ compared to the group with eGFR ≥ 90 ml/min/1.73 m^2^. There is a non-linear relationship between eGFR and 28-day all-cause mortality. This is consistent with the findings of Takashi Ui [20] et al. They showed that there was a U-shaped relationship between eGFR and 30-day mortality in 136,896 patients undergoing surgery for gastrointestinal malignancy. We further calculated the threshold inflection point using a two-piecewise regression model. There was a negative correlation between eGFR and 28-day all-cause mortality on the left of the threshold inflection point. It will illustrates risk of poor outcomes according clinically cut-off value of eGFR (57 ml/min/1.73 m^2^) which is very important for making of clinical decision.

It is not clear whether poor renal function is just a marker for severe AP or whether there is a causal associated with the increased mortality. To investigate whether eGFR is a real risk factor for all-cause mortality in AP, a thorough understanding of how the pathogenic factors affect mortality may help reduce mortality treatment targets. Several factors may be associated with high mortality in patients with lower eGFR, such as volume overload, electrolyte disturbances, metabolic acidosis, and the negative impact of uremic compounds. Haas et al. [19] suggested that patients with mildly reduced eGFR might reflect a sicker patient population, whereas in cases of severely reduced eGFR, the complexity of the treatment strategies will increase significantly. A recent study [18] proved that mortality and pancreatic necrosis were significantly higher in patients with increased serum creatinine levels and low eGFR in the first 24 h.

This study had several strengths. First, we used both Cox proportional hazard regression and restricted cubic spline curves to explore the nonlinear relationship between eGFR and 28-day all-cause mortality in patients with AP. Second, we combined existing literature, clinical judgment, and statistical adjustments to minimize the effect of confounders. Third, to verify the robustness of the results, we performed a subgroup analysis.

There are some limitations to our study. First, this is a retrospective and observational cohort study. Although we adjusted for confounding factors, residual unknown factors may bias the estimated association. In particular, the information on the etiology of acute pancreatitis was not supplemented, because there were many missing values about the etiology of acute pancreatitis in the database. Second, we are limited to the parameters collected in the database. The cause of death and the causative role of renal impairment could not be identified. It is difficult to identify a preexisting renal disease or acute renal function impairment due to AP. Third, only adults were included in our study, and the relationship in children with AP could not be determined.

## Conclusions

There was a non-linear association between all cause mortality and eGFR in patients with AP. The eGFR was negatively correlated with 28-day all cause mortality in AP when eGFR is less than the threshold inflection point.Participants with lower eGFR (< 30 ml/min/1.73 m^2^) were more likely to have higher all-cause mortality, and we hope that our study will encourage more research into this with larger sample sizes.

## Data Availability

The data that support the findings of this study are available from MIMIC-III (v. 1.4), which is an open and free database. Researchers can apply for permission to access it by completing a course known as Protecting Human Research Participants online.

## Abbreviations

eGFR: Estimated glomerular filtration rate
AP: Acute pancreatitis
KDIGO: The Kidney Disease: Improving Global
CKD-EPI: Chronic Kidney Disease Epidemiology Collaboration
ICU: Intensive care units
MIMIC-III: Medical Information Mart for Intensive Care version III
CECT: Contrast-enhanced computed tomography
MRI: Magnetic resonance imaging
ICD-9: International Classification of Diseases, Ninth Revision
SOFA: Sequential Organ Failure Assessment
SIRS: Systemic Inflammatory Response System
WBC: White blood cell count
BUN: Blood urea nitrogen
SD: Standard deviation
CI: Confidence interval
ROC curve: Receiver operating characteristic curve
